# Critical gaps in nanoplastics research and their connection to risk assessment

**DOI:** 10.3389/ftox.2023.1154538

**Published:** 2023-04-24

**Authors:** Brittany E. Cunningham, Emma E. Sharpe, Susanne M. Brander, Wayne G. Landis, Stacey L. Harper

**Affiliations:** ^1^ Department of Environmental and Molecular Toxicology, Oregon State University, Corvallis, OR, United States; ^2^ Institute of Environmental Toxicology and Chemistry, Western Washington University, Bellingham, WA, United States; ^3^ Department of Fisheries and Wildlife, Coastal Oregon Experiment Station, Oregon State University, Corvallis, OR, United States; ^4^ School of Chemical, Biological and Environmental Engineering, Oregon State University, Corvallis, OR, United States; ^5^ Oregon Nanoscience and Microtechnologies Institute, Corvallis, OR, United States

**Keywords:** microplastic, nanoplastic, risk assesment, toxicity, Bayesian network

## Abstract

Reports of plastics, at higher levels than previously thought, in the water that we drink and the air that we breathe, are generating considerable interest and concern. Plastics have been recorded in almost every environment in the world with estimates on the order of trillions of microplastic pieces. Yet, this may very well be an underestimate of plastic pollution as a whole. Once microplastics (<5 mm) break down in the environment, they nominally enter the nanoscale (<1,000 nm), where they cannot be seen by the naked eye or even with the use of a typical laboratory microscope. Thus far, research has focused on plastics in the macro- (>25 mm) and micro-size ranges, which are easier to detect and identify, leaving large knowledge gaps in our understanding of nanoplastic debris. Our ability to ask and answer questions relating to the transport, fate, and potential toxicity of these particles is disadvantaged by the detection and identification limits of current technology. Furthermore, laboratory exposures have been substantially constrained to the study of commercially available nanoplastics; i.e., polystyrene spheres, which do not adequately reflect the composition of environmental plastic debris. While a great deal of plastic-focused research has been published in recent years, the pattern of the work does not answer a number of key factors vital to calculating risk that takes into account the smallest plastic particles; namely, sources, fate and transport, exposure measures, toxicity and effects. These data are critical to inform regulatory decision making and to implement adaptive management strategies that mitigate risk to human health and the environment. This paper reviews the current state-of-the-science on nanoplastic research, highlighting areas where data are needed to establish robust risk assessments that take into account plastics pollution. Where nanoplastic-specific data are not available, suggested substitutions are indicated.

## 1 Introduction

**FIGURE 1:**
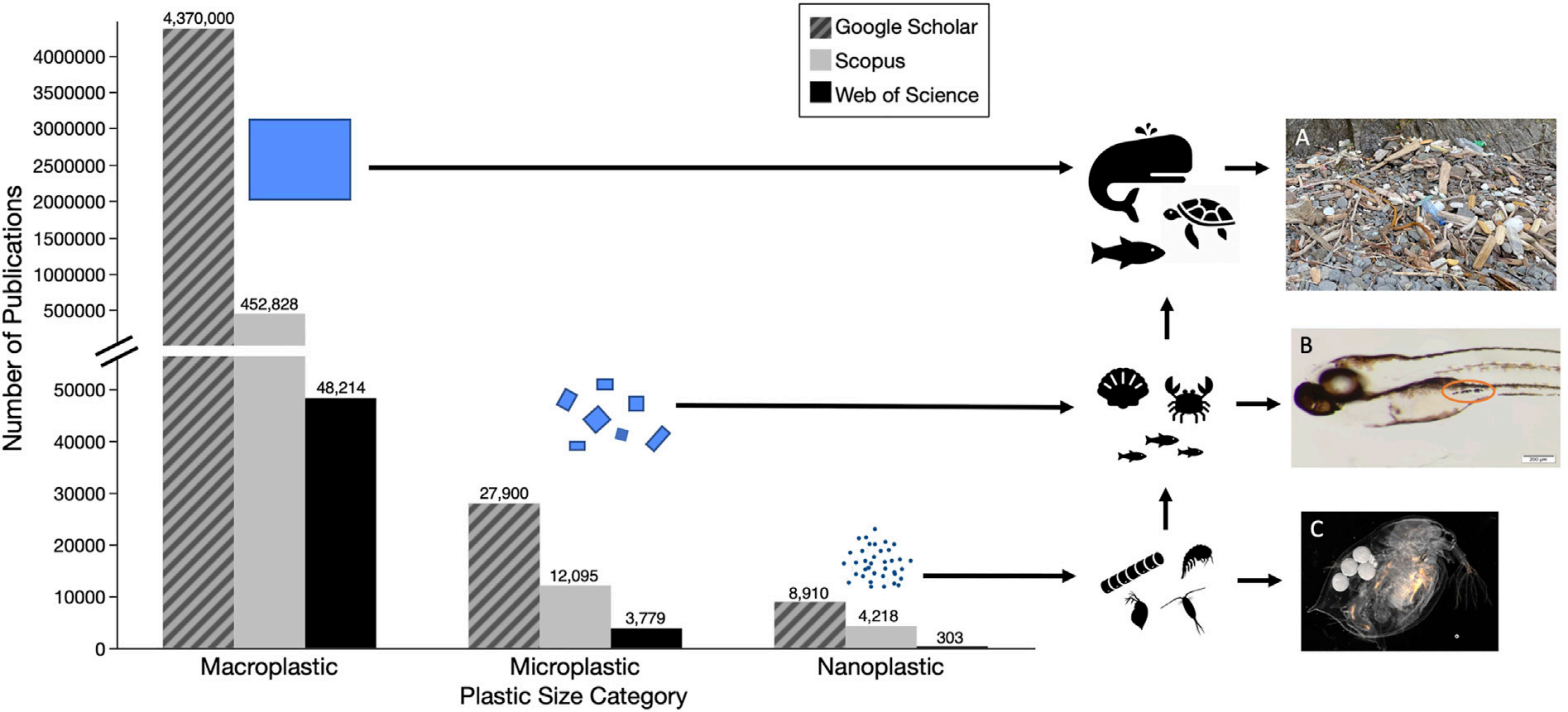
Numbers of papers on different size categories of plastic pollutants based on searches of three databases (i.e., Google Scholar—hatched grey, Scopus—grey, and Web of Science - black) **(A)** Macroplastic pollution washed up in the Katmai National Park, Alaska July 2021 **(B)** Exposed Zebrafish (1–20μm, tire particles) with ingested microplastics **(C)** Exposed *Daphnia* (40nm, fluorescent polystyrene). Detailed methods for literature search are described in Supplementary Material.

Plastic pollution, synthetic organic polymers that are resistant to degradation, is a worldwide issue that, while first noted in the 1970s (Carpenter and Smith, 1972; Colton et al., 1974), did not begin to be widely studied until the 2000s (Derraik, 2002). Annual production of plastics had grown to approximately 368 million tons in 2019 (PlasticsEurope, 2020) and is predicted to increase exponentially by 2050 (Jambeck et al., 2015). In the United States, plastics are currently classified as solid waste, though some have made the case for their classification as hazardous waste, based on established physical and chemical dangers they pose to organisms as well as their persistence in the environment (Rochman et al., 2013). Plastic debris has been observed in a variety of sizes and can be generally classified as macroplastics (>25 mm), microplastics (<5 mm), and nanoplastics (<1 μm) (Gigault et al., 2018). Whereas documentation and research initially focused on easily-visible macroplastics, recent years have witnessed a shift by researchers to smaller microplastics (<100 μm). Yet, comparatively less research has been done on the occurrence and fate of nanoplastics; indeed, many early reviews conducted on plastic pollution failed to mention particles this small (Derraik, 2002; Moore, 2008; Barnes et al., 2009; Ryan et al., 2009; Wabnitz and Nichols, 2010; Sigler, 2014; Li et al., 2016; Avio et al., 2017). Figure 1 illustrates the past focus of plastic research on macro and microplastic pollutants, and highlights the paucity of research on nanoplastics in the environment.

The primary reason for this knowledge limitation is that most methods of detection and identification developed for microplastics do not work for nanoscale particles (Renner et al., 2018; Brander et al., 2020; Caputo et al., 2021). As particle size decreases, plastics become difficult––if not essentially impossible––to detect in the environment. Nanoplastics originate from primary and secondary sources: (1) primary source nanoplastics are manufactured at the nanoscale for industrial, agricultural, and biomedical uses (ASHTON et al., 2010; Cole et al., 2011; Andrady, 2015), or are generated during the manufacture of polymers (Hernandez et al., 2017); (2) secondary source nanoplastics are derived from macro- and microplastics that break down into nano-sized particles in the environment (Lambert and Wagner, 2016; González-Pleiter et al., 2019). Limited knowledge exists about the introduction and transport of nanoplastics in the environment. For example, little is known about the probability and quantity of uptake by microscopic organisms and those higher up the food chain. Overall, both the quantity and effects of plastic pollutants may be underestimated. The chemical and physical properties of plastics are also critically important in understanding the breakdown, transformations and potential environmental impacts of nanoplastics. While there is mounting research on the effects of microplastics in organisms and the environment, there remains appreciable holes in nanoplastic research and how plastic particle fate and behavior changes as it moves from the micro-to the nano-scale. Existing knowledge of microplastic exposures and impacts, as well as research on non-plastic nanomaterials can be harnessed to inform the direction of future nanoplastic studies as well as risk assessment. These knowledge gaps are the focus of subsequent sections, followed by a discussion on their connection to risk assessment.

Ecological risk assessment (ERA) has been applied to a variety of scenarios (Landis, 2021). ERA is described as a cause-effect interaction between (1) sources, (2) stressors, (3) geographical location, and (4) effects and impacts described as probability distributions (Landis and Wiegers, 1997; Wiegers et al., 1998; Landis and Wiegers, 2007). Sources are the type of location for the types of activity that introduce specific stressors to the environment. These stressors can include chemical contaminants, nutrients, non-indigenous species, emergent diseases, and plastics. The stressors have the potential to change ecological systems, and can be biotic or abiotic. Location (often referred to as habitat) is the specific geographical place where the stressor and the endpoints interact. The effects describe the exposure-response relationships between the stressors and endpoints. In risk assessment, an endpoint is defined as a property of the ecosystem that is culturally valued, and is distinguished by an entity or attribute. For example, the endpoint of the spring run Chinook salmon (the entity), with the attribute as a population size at or above a management goal. If there are multiple stressors and multiple endpoints, the matrix of the probability distributions describing the effect to each endpoints is defined as the impact. Harris et al. (2017) has demonstrated that both ecological and human health endpoints can be evaluated applying the same process.

Nanoplastics can be evaluated in the same manner to estimate ecological risk. This manuscript describes and evaluates the current knowledge available to evaluate ecological risk using the described framework. The sources of nanoplastics are described and the routes of these materials to the environment. Nanoplastics can be introduced directly or the result of alteration of macro- and microplastics after introduction. The nanoplastics then exist in the environment by direct introduction or by transport processes and these are listed. There are several modes of toxicity that have been observed or hypothesized for these materials from molecular interactions to mechanical process. These interactions can lead to effects that are ecologically important, specifically those that affect survivorship, growth and reproduction that affect population and community dynamics. A carefully summary of the research and a critique of the state of the art is presented for each of these factors.

## 2 Detecting and estimating exposure to nanoscale plastics

**FIGURE 2:**
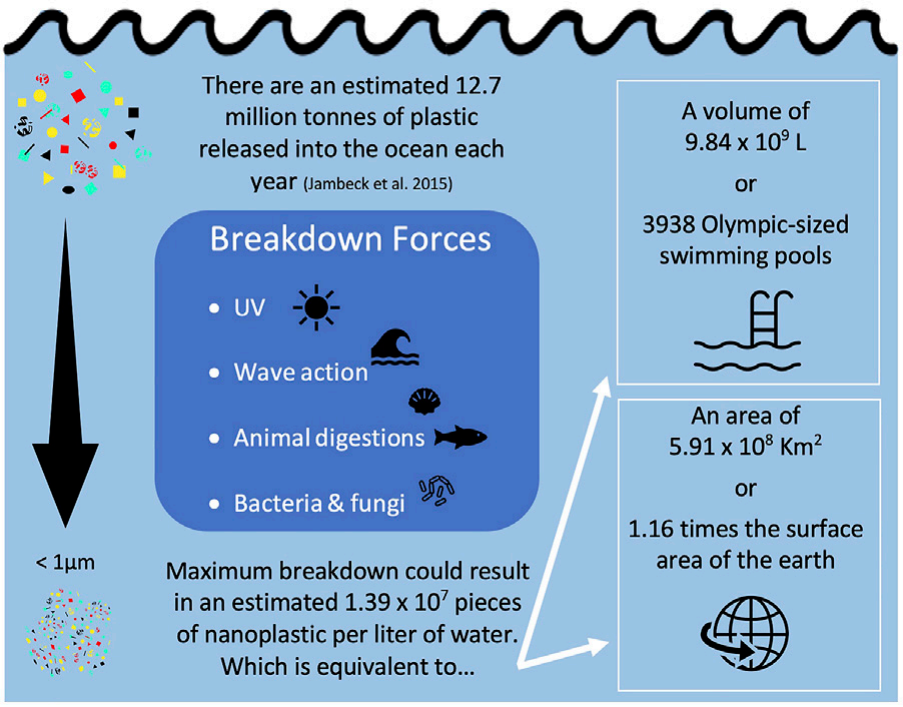
Calculation of equivalent nanoplastic particle count, volume, and surface area using a mass balance approach detailed in Supplemental spread sheet.

Nanoplastic exposure is difficult to quantify as we still do not have clear understanding of their concentrations and characteristics in the environment. In their opinion piece, Gigault et al. (2018) attempt to define the word nanoplastic, stating that nanoplastics only come from the breakdown of larger plastics. This statement ignores the primary sources of nanoplastics which are manufactured at a nano-scale. Some examples of this include nanoplastics made for 3D printing (Stephens et al., 2013), drug delivery (Hans and Lowman, 2002), and those used to encapsulate pesticides (Meyer et al., 2015). Any source of macro/micro plastic pollution, can also eventually give rise to nanoplastics through degradation (Andrady, 2015; Lambert and Wagner, 2016). For example, degradation of the estimated 5 trillion (5.0 × 10^12^) pieces of microplastic in the environment (Eriksen et al., 2014) could result in 5.0 × 10^14^ to 5.0 × 10^15^ pieces of nanoplastic, assuming all were degraded to the nano-scale. One study estimated that up to approximately 14 million tons of plastic were released into the ocean each year (Jambeck et al., 2015), which if entirely degraded to the nano-scale, would be equivalent to 1.52 × 10^7^ pieces of nano-sized plastics per liter of water (Figure 2). In a 2016 letter, Lenz et al. used observed environmental levels of microplastics, all larger than 5 µm, to project concentrations of nano-sized plastic particles, down to 0.05 µm, in the environment. At Lenz et al. (2016)’s smallest nano-size, the 95% confidence interval of their regression line spanned from approximately 1 × 10^3^—1 × 10^9^ particles per L, which is in line with the degraded concentration we calculated above for Jambeck et al. (2015). No other estimates have been done yet for the concentration of nanoplastics in the environment, or the projected size-ranges that these nanoplastics would fall into.

Once in the environment, nanoplastics can come in contact with organisms and be ingested, inhaled, and/or consumed. Because of their small size, nanoplastics are more likely to pass through biological membranes (Rossi et al., 2013), meaning they can be internalized in additional ways beyond what is possible for a larger-sized particle (Kolandhasamy et al., 2018). Humans are exposed to nanoplastics through a variety of sources including in the air breathed in, the use of personal care products, and consumption of contaminated water and seafood (Yee et al., 2021). However, due to the paucity of data in this area, researchers often rely on studies of microplastic exposure to infer nanoplastic exposure. Studies have shown that both consumption and inhalation are major routes of microplastic intake for humans (Cox et al., 2019; Mohamed Nor et al., 2021). Currently, human nanoplastic exposure has not been quantified because of the lack of information available on environmental nanoplastics; studies are limited by technological limits of plastic detection and identification at the nanoscale.

For larger particles, environmental and/or organismal plastic detection is conducted visually (Moser and Lee, 1992; Eriksen et al., 2014; Van Sebille et al., 2015; Forrest and Hindell, 2018; Kotar et al., 2022). Additional methods and plastic detection and quantification include: Raman Spectroscopy, Fourier Transform Infrared Spectroscopy (FTIR), and Pyrolysis-gas chromatography coupled to mass spectrometry (Bouwmeester et al., 2015; Renner et al., 2018; Primpke et al., 2020; Cowger et al., 2021). Because the current limits of detection for plastic particles is approximately in the 1 µm range for µRaman and the 201 µm for µFTIR (Bouwmeester et al., 2015; Renner et al., 2018; De Frond et al., 2023), these methods fail to detect most nanoplastics. Therefore, existing estimates of plastic concentrations in the environment likely underestimate the true value. Currently, neither Raman nor FTIR are used to identify the majority of nanoplastics because of the particle mass necessary for detection. However, advances are being made to expand these limits of detection. Though surface enhanced Ramen spectroscopy (Sobhani et al., 2020; Zhou et al., 2021) and pyrolysis (Ter Halle et al., 2017) have been used to identify polymers in the nano size-range, pyrolysis cannot be used for visualization and quantification of individual particles. Additionally, pyrolysis efficiency is not the same for all types of plastics (Ter Halle et al., 2017); for example, Hermabessiere et al. (2018) found that temperature had a large impact on the signal and types of products of pyrolysis for various plastics. Dynamic light scattering (DLS) and scanning electron microscopy (SEM) have also been used to confirm the presence of nanoplastics in samples (Hernandez et al., 2017), but these operations cannot identify the chemical composition of the particles. A problem with many of these common methods of plastic detection and identification is that as the particle size of the plastic decreases, as with nanoplastics, the probability of misidentification increases (Bouwmeester et al., 2015; De Frond et al., 2023). Most recently, enrichment of environmental samples has been suggested to quantify nanoplastics in water (Cai et al., 2021) and soil (Wahl et al., 2021) samples.

## 3 Fate and transport of nanoplastics

### 3.1 Fate and transport of nanoplastics based on microplastics

Plastic particles, especially those in smaller size-ranges, have been found in almost all environmental compartments ranging from the open ocean to farmlands to polar regions (Barnes et al., 2009; Cózar et al., 2014; Van Sebille et al., 2015; Ng et al., 2018; Obbard, 2018; Ross et al., 2021). Even in remote, unpopulated areas of the world, studies have detected quantities of environmental plastics comparable to those in large cities (Allen et al., 2019; Brahney et al., 2020). Though we are beginning to gain an understanding of the transport of microplastics through the air and water, little information exists on how these same mechanisms may transport nanoplastics. Common characteristics shared between micro- and nanoplastics, such as polymer charge and density, make it reasonable to conjecture that many of the mechanisms of transport for larger plastic particles are also responsible for transport of nanoplastics (Table 1). For example, plastics are known to be transported in ocean currents (Avio et al., 2017), sewer systems (Sun et al., 2019), and in the wind (Ryan et al., 2009; Brahney et al., 2021) and atmosphere (Allen et al., 2019). However, it cannot be assumed that fate and transport will be exactly the same between plastics with different sizes or morphologies. Nanoplastic debris is known to behave differently than microplastics (Gigault et al., 2021), especially in terms of floatability (Ter Halle et al., 2017).

**TABLE 1:** What we can and cannot draw from microplastics and nanomaterials research to inform nanoplastics risk assessment.

	Microplastics	Nanomaterials
**EXPOSURE**		
**Environmental Transport:**	The density of microplastics and nanoplastics would be the same when broken down from the same macroplastic material. However, nanoplastics may remain suspended in the water column because their mass is so small that gravity does not affect them	Transport through the environment will depend upon the agglomeration state of the nanomaterials and nanoplastics. Both processes should be affected by salinity, pH and organic matter. Transport could be similar if the nanomaterials are similar to the density of the nanoplastics (e.g., nanocellulose)
**Environmental Fate:**	Fate may be similar if nanoplastics agglomerate into micron-sized agglomerates. Agglomeration is more likely in higher salinity environments	Environmental fate may be comparable if the nanomaterials and nanoplastics have similar densities (e.g., nanocellulose or lignin). The formation of an organic matter layer on the surface of nanomaterials and nanoplastics would be the same if the outermost surface chemistry is the same. Nanoplastics are persistent and do not dissolve; whereas, some nanomaterials (i.e., transition metals) readily dissolve unless coated with a protective barrier
**Environmental Sampling:**	Current collection techniques for microplastics do not retain nanoplastics so no environmental quantification has been done for nanoplastics	The same issues in environmental sampling for nanomaterials and nanoplastics including difficulties collecting and concentrating the samples, interference of other colloids in the system, and locating them in a complex matrix
**Identification:**	The same composition of materials found at the macro scale translate to compositions found at the micro scale. It is likely, but not verified, that those will also translate to composition found at the nanoscale	Most engineered nanomaterials are produced at the nanoscale and are therefore considered primary particles. Whereas, only a very few types of nanoplastics (e.g., PS, PMMA) are generated as primary particles, most result from the breakdown of microplastics. Standardized reference materials are available for some classes of nanomaterials, but remain lacking for nanoplastics. Dynamic light scattering and nanoparticle tracking analysis can be used for both nanoplastics and nanomaterials. Since both instruments rely on the assumption that the particles are spherical, the vast majority of nanoplastics will violate that assumption while many engineered nanomaterials are engineered to be spheres
	Most micro and nanoplastics in the environment are from the breakdown of larger plastics and considered secondary particles. Lack of standard reference materials for both micro and nanoplastics. FTIR and pyrolysis-GC-MS can be used to identify the type of microplastics; however, nanoplastics do not have enough mass for these instruments/techniques to work. Shape can be determined for microplastics using a dissecting or compound microscope; whereas, nanoplastics require the use of electron microscopy to determine shape	
**Quantification:**	Environmental concentrations can be extrapolated across size classes. The relative concentrations of macroplastics found at the macro scale translate to the relative concentrations found at the micro scale. It is likely, but not verified, that those ratios of different types of microplastics are comparable to the ratios found at the nanoscale	Instruments used to measure the size, zeta potential and agglomeration state of nanomaterials can be applied for nanoplastics. For example, dynamic light scattering and nanoparticle tracking analysis can be used to assess nanoplastics; however, clear plastics sometimes evade detection so staining may be required prior to measurement
**EFFECTS**		
**Who is exposed:**	Larger organisms can take up micro and nanoplastics incidentally, and microplastics actively. Smaller organisms that have taken up smaller microplastics and nanoplastics may serve as another source for larger organisms through trophic transfer. Filter feeders are more likely to take up micro and nanoplastics when they are stabilized in suspension	Because of their small size, bulk forces like gravity have little effect on nanomaterials including nanoplastics. If unagglomerated, both can remain suspended in water bodies indefinitely leading to exposure of organisms the live in, or traverse, the water column. Small and large organisms can incidentally or actively ingest nanomaterials and nanoplastics
**Uptake/Translocation:**	The uptake and translocation potential of micro and nanoplastics will differ. While microplastics may be taken up by phagocytlosis, nanoplastics can be actively taken up by cells and translocate into inner body tissues and organs. These uptake mechanisms are size dependent and more can be gleaned from other nanomaterials *versus* microplastics	The mechanisms of uptake would be similar between nanomaterials and nanoplastics, particularly when uptake is a function of size. For example, particles around 100 nm can enter via clathrin-mediated endocytosis, while those between 50 and 80 nm can enter via caveolae-mediated endocytosis
**Biodistribution:**	The biodistribution of microplastics will be limited by their size where only the smaller microplastics will be small enough to enter inner tissues and organs. Nanoplastics are known to translocate to the liver after ingestion	Biodistribution should be similar among nanomaterials and nanoplastics that have similar size, shape and charge. Accumulation of nanoplastics could occur in the lysosomes as the materials are difficult to break down even in an extremely low pH environment
**Toxicity:**	Reactive oxygen species generation, oxidative stress, inflammation and metabolic disruption have been indicated for both micro and nanoplastics	Reactive oxygen species generation is a predominant finding for both nanomaterials and nanoplastics. Particle specific effects would be expected for both nanomaterials and nanoplastics. Inflammation, oxidative stress and metabolic disruption have also been indicated for both nanoplastics and nanomaterials
**UNKNOWNS**		
**Reference materials:**	Literature is growing on the development of reference materials for the study of microplastics and the US National Institute of Standards and Technology has a MNP Metrology Project underway to establish standardized methods for size-based separations from complex matrices, chemical characterization protocols, and test materials necessary to enable quantification of micro and nanoplastics. Hawaii Pacific University offers a reference materials kit that has been shared across the micro and nanoplastics field. Reference materials are being generated for tire tread as well. Commercially available reference materials for nanoplastics remain limited to spherical polystyrene, polyethylene and polymethyl methacrylate	Many engineered nanomaterials are commercially available or can be synthesized in small batches that can be strategically designed to tweak one physicochemical property to investigate the impact of that parameter on the behavior of the nanomaterial. Nanomaterials can be precisely engineered for size, shape and surface chemistry and are available in homogeneous suspensions. Whereas, only a few nanoplastics (e.g., polystyrene, PMMA) are commercially available and they are all spherical which is not representative of what is found in the environment from the breakdown of macro and microplastics. In addition, the surface chemistries available on purchased nanoplastics include amine (+) or carboxyl (-) groups, or could be left neutral with no surface chemistry added
**Dose metrics:**	Microplastics dose is often reported as mg/L as that is the easiest dose metric to empirically measure. Nanoplastics dose; however, is more often reported as particle #/L. Conversion calculations have been established by Besseling et al. (2014), but are still not routinely used	For nanomaterials that dissolve, surface area is the best dose metric to apply since it determines the rate of dissolution. Most other nanomaterial toxicity studies report a mass-based dose. Since nanoplastics do not dissolve, the number of particles is typically reported although this has not been standardized. There are conversion equations to convert between mass and particle number; however, there are many assumptions that are likely violated in this conversion
**Requirements for decision makers:**	Policies aimed at microplastics can aid in decreasing secondary shed of nanoplastics, but would not address primary nanoplastic production	Structure-activity relationships determined for nanomaterials could likely be applied to nanoplastics with the same surface chemistry, particularly for biopolymeric nanomaterials such as lignin or cellulose
**K**_**ow**_ **:**	The octanol/water partition coefficient (K_OW_) is one important consideration when asking if plastic particles are acting as vectors for other contaminants and compounds	The octanol/water partition coefficient (K_OW_) remains a challenge to assess for both nanomaterials and nanoplastics

**FIGURE 3:**
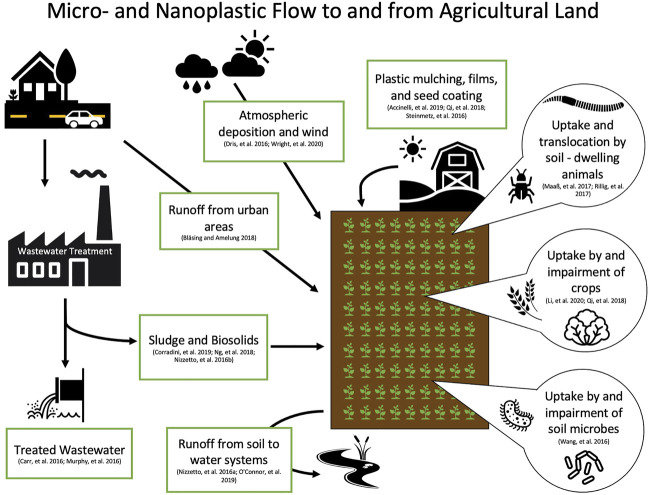
Diagram of potential sources and flow of micro- and nanoplastics to soil, and uptake by plants and soil-dwelling organisms (Accinelli et al., 2019; Bläsing and Amelung, 2018; Carr et al., 2016; Corradini et al., 2019; Dris et al., 2016; Li et al., 2020a; Murphy et al., 2016; Nizzetto et al., 2016a; O'connor et al., 2019; Qi et al., 2018; Rillig et al., 2017; Steinmetz et al., 2016; Wang et al., 2016; Wright et al., 2020).

Because the majority of plastic research has focused on aquatic environments, we can make even fewer assumptions about the transport and fate of nanoplastics in terrestrial environments. One of the known contributors of plastic particles to soil is through the application of biosolids. Nizzetto et al. (2016b) estimated that between 48 and 330 thousand tons of microplastics contained in biosolids are added annually to farmlands in the United States. In a different study on Canadian biosolid samples, concentrations of microplastics up to 1.4 × 10^4^ particles per kg were detected (Crossman et al., 2020). However, the plastic particles in the biosolids do not remain contained within the agricultural lands; Crossman et al. (2020) also found that over 99% of these micro-sized particles were further transported into aquatic environments. Furthermore, Geyer et al. (2022) evaluated microfiber pollution and estimated that in 1 year 1.6 kilotons of microfibers were released into terrestrial environments in California, United States alone. They also modeled the removal of microfibers from wastewater, and found that their redirection into biosolids resulted in increased application to terrestrial environments (Geyer et al., 2017; Geyer et al., 2022). Though biosolids have not yet been evaluated for concentrations of nano-sized plastics, the number of nanoplastics in farmlands may be higher considering additions of fragmented waste from other sources (Ng et al., 2018). Furthermore, earthworms have been shown to fragment microplastics into nanoplastics (Kwak and An, 2021). This emphasizes the urgency for research on nanoplastics in terrestrial environments, as farm soils in particular may act as critical pathways of plastic fragmentation and exposure to both organisms and humans (Hurley and Nizzetto, 2018) (Figure 3).

### 3.2 Fate and transport of nanoplastics based on non-plastic nanoparticles

Nanomaterial research can be useful in informing study of nanoplastics, particularly in relation to the suspension of nano-scale particles. As nanoparticles have different properties than their bulk counterparts (Farré et al., 2009), we cannot assume that nanoplastics will behave in the same manner as larger plastics. For example, nanoparticles are known to form agglomerates, which can affect their dispersion. The rate and strength of this agglomeration depends on the physical and chemical characteristics of the media that they are in (Gigault et al., 2018; Wu et al., 2019b; Shupe et al., 2021). At the nanoscale, Brownian motion can affect particle sedimentation (Hassan et al., 2014). Therefore, it cannot be assumed that nanoplastics will be transported and partitioned in the same manner as macro- or microplastics. While studies on non-plastic nanomaterials can inform future research on nanoplastics, we cannot simply assume that environmental characteristics and behaviors will be identical (Table 1). Nanoplastics can differ from these better studied nanomaterials in several ways including: (1) a lack of aqueous solubility (Wagner et al., 2018), (2) a lack of exterior charge (Rist and Hartmann, 2018), and/or (3) low density (Rist and Hartmann, 2018). The partitioning of nanoplastics is further complicated by the fact that their transport in differing media is partially dependent on both the particles surface modifications and transformations occurring in the environment (Dong et al., 2019).

## 4 Current state of nanoplastic research

### 4.1 Uptake and absorption

Organisms have been shown to uptake nanoplastics rapidly, although the mechanisms of nanoplastic uptake are not fully understood (Kashiwada, 2006; Casado et al., 2013; Booth et al., 2016; Pitt et al., 2018a; Brandts et al., 2020). While the majority of organisms likely ingest nanoplastic particles, either intentionally or unintentionally during feeding (Ward and Kach, 2009), other modes of uptake, such as inhalation, are possible as well (Lim et al., 2021). In fact, one study suggests that in addition to ingestion, bivalves might internalize plastic particles through adherence (Kolandhasamy et al., 2018). The chemical properties of the nanoplastics play a role in determining particle uptake and tissue distribution, which is why nanoplastics will not necessary behave in the same manner as other manufactured nanoparticles. For example, comparison of nanoparticle distribution in *Pecten maximus* exposed to polystyrene (PS) and Ag nanomaterials of similar sizes showed that the particles accumulated in different organs in the scallops (Al-Sid-Cheikh et al., 2013; Al-Sid-Cheikh et al., 2018). For plastic with surface charge modifications, charge seem to be a central component in determination of uptake and dispersion into tissue. Negatively and positively charged nanoplastics of the same type have been found to have different levels of uptake (Cole and Galloway, 2015), accumulation (Sun et al., 2020), and toxicity (Bergami et al., 2017), as well as different sites of accumulation within the organism (Bergami et al., 2016).

Nanoplastic particle size also plays a role in both rate of uptake and tissue distribution. Studies using rotifers, larval oysters, scallops, zebrafish, and *Daphnia* have found that in general smaller particles are internalized faster and disperse throughout the organism, while larger particles are taken up more slowly and tend to accumulate in the intestinal tract (Snell and Hicks, 2011; Cole and Galloway, 2015; Lu et al., 2016; Al-Sid-Cheikh et al., 2018). However, a couple of studies, also using *Daphnia*, contradict these results, citing a greater intake of larger particles than smaller particles (Rosenkranz et al., 2009; Rist et al., 2017). One reason for this discrepancy is that though these studies showed higher uptake of the larger particles, when they compared surface areas they found that values were actually higher for the intake of the smaller than the larger particles (Rosenkranz et al., 2009; Rist et al., 2017). Another reason may be related to the aggregation of certain nanoplastics. Uptake of smaller nanoplastics may have been higher in certain studies because of their increased tendency to form agglomerates resulting in an overall larger size (Ward and Kach, 2009). Nanoparticle size has also been shown to impact the location of particle accumulation within an organism (Lee et al., 2019). Another physical component that likely plays an important role in the determination of nanoplastics uptake is particle morphology or shape, though no studies have investigated this to date.

Due to the above-mentioned difficulties of locating nanoplastics, many uptake studies used commercially-available fluorescent, spherical PS nanoparticles. For example, several studies demonstrated the movement of fluorescence from the GI tract into lipid droplets in *Daphnia* (Rosenkranz et al., 2009; Brun et al., 2017; Cui et al., 2017). However, these fluorescent nanoplastics have since been demonstrated to actually leach fluorescence (Catarino et al., 2019; Schür et al., 2019). Furthermore, toxicity has been found to decrease, or even disappear, following dialysis of nanoplastics (Heinlaan et al., 2020). This information put into question the results of many of those studies and the true ability of various sizes and exposures of nanoplastics to transverse the gut. Yet, the ability of some nanoplastics to disperse into tissues has been demonstrated in studies not using fluorescent nanoplastics, suggesting that they can in-fact cross epithelial walls and cell membranes (Rossi et al., 2013). For example, nanoplastic uptake and partial depuration has been demonstrated in *Gammarus pulex* using metal-doped nano PS (Redondo-Hasselerharm et al., 2021). Overall, further uptake and distribution assessments are needed––specifically studies that use either non-fluorescent or dialyzed fluorescent nanoplastics, as well as nanoplastics of different morphology, sizes, and polymer types, to verify the true fate of these particles within an organism.

### 4.2 Biodistribution and effects

#### 4.2.1 Impacts of PS nanoplastic on aquatic organisms

The effects of PS nanoplastics have been assessed using a variety of aquatic life-forms. Many studies have investigated the effects of PS nanoplastic on *Daphnia*. Exposure of *Daphnia* to nanoplastics can impact survival, growth, reproduction, and metabolic and immune functions (Besseling et al., 2014; Brun et al., 2017; Cui et al., 2017; Liu et al., 2020b; Liu et al., 2021). Additionally, differing magnitude of effect for *Daphnia pulex* exposed at different ages suggests that age may be an important factor affecting the toxicity of nanoplastic (Liu et al., 2018). Bacteria exposed to PS nanoplastic showed decreased cell growth and increased intracellular reactive oxygen species (Sun et al., 2018). Additionally, PS nanoplastic exposure was associated with decreased growth rates in green algae, *Dunaliella tertiolecta* (Sjollema et al., 2016; Bergami et al., 2017) and *Scenedesmus obliquus* (Besseling et al., 2014). Pinsino et al. (2017) report that sea urchin, *Paracentrotus lividus*, embryos exposed to nanoplastic (PS-NH_2_, original particle size 50 nm, agglomerate ∼143 nm) displayed developmental malformations and increased expression of stress proteins. Balbi et al. (2017) exposed mussel (*Mytilus galloprovincialis*) embryos to 50 nm cationic polystyrene (PS-NH_2_) and found that embryos exposed to the plastics both developed abnormally and exhibited altered expression of genes related to shell formation (Balbi et al., 2017). In adults, exposure to nano PS had negative effects on mussel immune cells (Sendra et al., 2020a; Sendra et al., 2020b) and altered feeding behavior (Wegner et al., 2012). Furthermore, PS nanoplastic exposure has been shown to cause mortality in brine shrimp (*Artemia franciscana*) larvae (Bergami et al., 2017), and to affect feeding, behavior and physiology of adults (Bergami et al., 2016). PS nanoplastics are known to accumulated in the tissue of larval zebrafish (Pitt et al., 2018a). Neurotoxicity and oxidative damage are commonly observed effects in zebrafish nanoplastic exposures (Sarasamma et al., 2020; Sökmen et al., 2020), and nanoplastics have been found to impact zebrafish behavior even when microplastics have not (Chen et al., 2017). Though an abundance of information exists on the impacts of nanoplastic PS spheres, these particles are not representative of the full diversity of nanoplastic pollution in the environment, and their impacts cannot simply be extrapolated to nanoplastics of differing compositions.

#### 4.2.2 Impacts of non-PS nanoplastic on aquatic organisms

Far fewer studies have investigated the effects of organismal exposure to nanoplastics other than PS. One study demonstrated that short-term exposure of a marine fish, *Dicentrarchus labrax,* to 45 nm PMMA nanoplastics effected lipid metabolism and impaired immune function (Brandts et al., 2018). For plankton, PMMA nanoplastics also inhibited growth in *Rhodomonas baltica* (Gomes et al., 2020) and caused oxidative stress in *Gymnodinium aeruginosum* (Huang et al., 2021). Additionally, exposure of cnidarian, *Hydra viridissima*, to nano PMMA resulted in mortality and malformations (Venâncio et al., 2021). Greven et al. (2016) found that exposure to polycarbonate (PC, 158.7 nm) nanoplastics had effects on the immune system of freshwater fish, *Pimephales promelas*. Additionally, *Mytilus edulis L*. exposed to high-density polyethylene (HDPE) particles exhibited an inflammatory cellular response (Von Moos et al., 2012). Further, González-Pleiter et al. (2019) assessed the effects of polyhydroxybutyrate (PHB) nanoplastics on cyanobacteria (*Anabaena*) green algae (*Chlamydomonas reinhardtii*), and *Daphnia* (*Daphnia magna*) and found an increase in ROS formation in all organisms. Clearly, non-PS nanoplastics carry unique potentials for toxicological impacts and their full impacts are not captured in nano-PS studies alone.

#### 4.2.3 Impacts of nanoplastic on terrestrial organisms

There is a general lack of research on the effects of micro- and nanoplastic on terrestrial organisms (Gomes et al., 2022). The limited studies that do exist for these organisms show that, as with aquatic organisms, plastic exposure can be detrimental to growth and development. For example, for soil oligochaete *Enchytraeus crypticus,* exposure to PS nanoplastics resulted in a shift in their microbiome leading to a decrease in weight and reproduction (Zhu et al., 2018). Nano PE was also found to alter the gut microbiome of earthworm (*Metaphire vulgaris*) (Wang et al., 2021). Though far less studied, plants have been shown to accumulate (Sun et al., 2020) and be effected by (Li et al., 2020b) nanoplastics in soil as well. There are still relatively few publications on the toxicity of nanoplastic particles for mammals. For Wistar rats, ingestion of PS nanoplastics has been shown to have neurobehavioral consequences (Rafiee et al., 2018) and induce oxidative stress (Babaei et al., 2021). Additionally, exposure of mice to nano PS resulted in impacts to their microbiome (Szule et al., 2022).

Additionally, though toxicity of plastics for humans has not been assed at the whole-organism level, the effects of PS nanoplastics has been investigated for a variety of human cell lines, including, but not limited to, colon, lung, epithelial, and bladder (Wu et al., 2019a; Lim et al., 2019; Poma et al., 2019; Xu et al., 2019). The micro- and nanoplastics, ranging in size from 20 to over 1,000 nm, have been shown to be readily taken up by the cells and result in alterations to gene expression (Lehner et al., 2019). Much of the literature on the toxicity of nanoplastics to human cells has focused on PS spheres (Wu et al., 2019a; Lim et al., 2019; Poma et al., 2019; Xu et al., 2019). However, Magri et al. (2018) created PET nanoplastics (<100 nm) and found that they were taken up by, and translocated across the epithelium layer of an intestinal model (MAAS et al., 2017; Magrì et al., 2018). Still, little is known about how effects on human cells may differ for nanoplastics composed of materials beside PS, or in other shapes.

#### 4.2.4 Impacts of nanoplastic morphology

The current focus on just one plastic type and shape (i.e., spherical PS) in nanoplastic ecotoxicity testing ignores hundreds of potential combinations of polymer type and particle morphology. We know that the toxicity of microplastics can vary with differing physical characteristics of the plastics; for example, Gray and Weinstein (2017) exposed grass shrimp to different sizes of spheres, fragments, and fibers, discovering a significant difference in the number of microplastic particles internalized between the different shapes. Additionally; Frydkjær et al. (2017) noted that *D. magna* egression of microplastics was slower for irregularly shaped fragments than for spheres. For zebrafish, the shape of the microplastic affects the accumulation and gut toxicity with fibers and then fragments being more toxic than beads (Qiao et al., 2019). A recent synthesis paper suggests that smaller microplastic particles cause greater toxicity (Thornton-Hampton et al., 2022). Nanoplastics also exist in a multiplicity of morphologies, a characteristic which the microplastic research tells us contributes to the toxicity of the particle. A comparison of the toxicity of nanoplastics of different shapes has not been done; however, some have assessed the impacts of nanoplastics in a fragment morphology. Both Siddiqui et al. (2022) and Cunningham et al. (2022) reported that nano tire fragments were highly toxic to aquatic organisms. This connection between morphology and toxicity is one reason that it is important to collect data on a variety of nanoplastic polymers and morphologies.

### 4.3 Elimination pathways and bioaccumulation

While studies have begun to document internalization of nanoplastics, very little is known about organisms’ abilities to expel these materials. Research suggests that as the size of the particle decreases so too does the organism’s ability to remove them from the body (Rist et al., 2017). Suspension feeding bivalves, mussels and oysters, had longer gut retention for nano-sized PS particles, than for microplastics (Ward and Kach, 2009). This trend was also observed in aquatic invertebrate, *Diaphanosoma celebensis,* as well (Yoo et al., 2021). However, within the size-category of nanoplastics, there is not a consensus on the size of particle that is egressed most quickly. There is a need for research on nanoplastic egression rates and percent retention. One limitation of existing studies is that in general, they each only compared two particles sizes, and that few of them were the same two sizes (e.g., 2 μm and 100 nm (Ward and Kach, 2009; Rist et al., 2019), 24 nm and 250 nm (Al-Sid-Cheikh et al., 2018), and 100 nm and 30 nm (Rist et al., 2017)). A comparison between only two particle sizes makes it difficult to draw conclusions about how patterns of ingestion, translocation, and egression may change as size changes. Additionally, the seemingly random sizes chosen for these exposures hampers comparison between studies.

In most cases organisms are not able to egress 100% of the nanoplastics they internalize. This may be due to the small size of nanoplastics, which increases their ability to cross biological membranes within organisms and/or to become entrapped (Rossi et al., 2013). Bhargava et al. (2018) exposed larval barnacles to nano PMMA and found that even following a single acute exposure over 3 h, the nanoplastics remained in the body of the barnacles after 7 days; they also observed accumulation of PMMA from chronic exposure (Bhargava et al., 2018). This capability to accumulate within an organism’s tissue enhances the possibility for trophic transfer and biomagnification. Casado et al. (2013) studied the effects of polyethyleneimine polystyrene nanoplastics on organisms at a variety of trophic levels including: algae (*Pseudokirchneriella subcapitata*), crustaceans (*Thamnocephalus platyurus* and *D. magna*), bacteria (*Vibrio fischeri*), and rainbow trout (*Oncorhynchus mykiss*) cell lines, . but did not identify trends between toxicity and trophic level of the exposed organism. In addition, a couple of trophic transfer studies have investigated the effects of PS nanoplastics on a simple food chain made up of green algae, zooplankton (*Daphnia*), and fish (carp). Cedervall et al. (2012) found that 25  nm PS nanoplastics were taken up by the algae and transported through the *Daphnia* to the fish. The nanoplastics accumulated in the fish and resulted in disruption of lipid metabolism and alterations in fish behavior (Cedervall et al., 2012). This was supported by Mattsson et al. (2017), who also demonstrate that nanoplastics transfer up a food chain from algae to zooplankton to fish. The PS particles that the fish acquired from their prey, crossed the blood-brain barrier and resulted in brain damage and altered fish behavior (Mattsson et al., 2017). Similarly, Chae et al. (2018) demonstrated that nano PS was transferred from alga, to *Daphnia*, to secondary-consumer fish (*Oryzias sinensis*), and finally to end-consumer fish (*Zacco temminckii*), and eventually impacted the swimming behavior of the fish. These studies suggest that nanoplastic exposure, either directly or through prey items, can result in behavioral disturbances that may have effects that ripple through the food chain. This emphasizes the need to investigate nanoplastic impacts at an ecosystem level.

### 4.4 Chronic and multi-generational exposures

The vast majority of studies on nanoplastic toxicity, accumulation, and transfer between organisms have been limited to acute or short-term exposures. Because most of the toxicity studies on nanoplastics use single-generation acute exposure, there remains large data gaps in the areas of chronic and multigenerational nanoplastic exposures. Chronic exposures likely more closely mirror the environmental conditions organisms experience. A few studies have investigated the chronic effects of PS nanoplastics on *Daphnia*. Zhang, et al. (2020) exposed *D. pulex* to 75  nm PS for 21 days and found a decrease in the expression of several key genes related to growth and reproduction. Additionally, they saw a change in sex ratio skewed toward an increase in male neonate (Zhang et al., 2020). Lui, et al. (2019) also exposed *D. pulex* to 75  nm PS for 21 days and reported dose- and time-dependent effects. They found that chronic exposure to the nanoplastics decreased reproductive fitness and increased expression of stress-response genes (Liu et al., 2019). Kelpsiene et al. (2020) exposed *D. magna* to positively charged (aminated) or negatively charged (carboxylated) PS nanoplastics for 103 days and observed a charge-specific decrease in survival rate. Chronic exposure to nanoplastics has also been investigated in mussel *M. galloprovincialis*, where 21-day exposure to PS resulted in genotoxicity and oxidative stress (Gonçalves et al., 2022). Additionally, 1 month exposures to PS nanoplastics have shown negative effects in adult zebrafish (Sarasamma et al., 2020). Overall, because of their short maturation time and life-span, chronic studies have focused on *Daphnia*; however, future studies will need to expand the evaluation of chronic effects of nanoplastics on a wider variety of organisms from differing trophic levels and ecosystems (e.g., freshwater, marine, terrestrial).

There is also a need to better understand the ability of nanoplastics to be passed from exposed parents to offspring, and the potential effects of this transfer on future generations. In two-generation chronic toxicity tests, Lee et al. (2013) showed that PS nanoplastic ingested by copepod (*Tigriopus japonicus*) resulted in increased mortality in the next-generation. Pitt et al. (2018b) exposed zebrafish to 42  nm PS, and found that the nanoplastics accumulated in various tissues of the F1 generation and had an effect on the larval antioxidant system. For organisms with different life histories, other methods of exposure during embryo development are possible. In *D. magna* exposed to 25  nm PS; Brun et al. (2017) reported that the nanoplastics accumulated in the lipophilic cells of embryos in the open brood pouch (Brun et al., 2017). Liu et al. (2020a) noted reproductive effects in the offspring of nanoplastic-exposed *D. pluex* parents. Additionally, Liu et al. (2020c) exposed *D. pluex* to PS nanoplastics for three generations and found alterations in gene expression. Parental exposure of *Caenorhabditis elegans* to nano PS was found to result in transgenerational toxicity (Sun et al., 2021). Though research in this area is expanding, much is left unknown about the potential for developing organisms to be exposed to nanoplastics and its effects on subsequent generations, particularly for organisms beyond *Daphnia* and exposures with non-PS nanoplastics.

## 5 Prioritized needs and a path forward with risk assessment

### 5.1 Risk assessment

There has been increased interest from the scientific community and the general public in characterizing and managing the potential risks of plastic particles, including nanoplastics, in the environment. In the terminology of the science of risk analysis, “risk” is defined as the probability of an effect on one or more specific endpoints due to a specific stressor or stressors (National Academies of Sciences and Medicine, 2016). A risk assessment determines the probability distribution of how often a specific change in the environment will affect something of value to society, such as human health, outdoor recreation, or the survival of a key species. This definition implies that there is a cause-effect chain of interactions between the input of the material into the environment and the alteration of the endpoint beyond the cultural goals of the society. The mere potential to cause an effect is not risk.

Ecological risk assessment is a process for quantitatively modeling the numerous interactions between stressors and endpoints and incorporating epistemic and other types of uncertainties (National Academies of Sciences and Medicine, 2016; Landis, 2021). Quantitative risk assessments can indicate where gaps lie in the data necessary to accurately estimate risk, therefore determining and ranking the priority of research needs. The initial steps of a risk assessment are to select ecological endpoints based on site-specific management goals and valued ecosystem services and to build a conceptual model for the relationship between stressors and endpoints. Next, spatial, toxicological, and ecological data are collected, and statistical tools and quantitative models are employed to characterize the causal relationships involved in the system and the potential to the endpoints. Finally, results from the assessment are communicated to key stakeholders and decision-makers who then make decisions about risk mitigation and management strategies (Landis et al., 2017; Coffin et al., 2022; Mehinto et al., 2022).

Here, a specific evaluation of previous risk assessments is made and the methods and results described (Sharpe, 2022). To date, these are examples of assessments for microplastics, not nanoplastics, but serve as examples. Finally, a proposed structure for a general approach to the risk assessment of nanoplastics is described. This approach is based on that developed for microplastics but with a focus on the particular issues when estimating risk due to nanoplastics.

### 5.2 A proposed nanoplastic risk assessment framework

An ecological risk assessment for nanoplastics requires a framework that: 1) considers the complex nature of plastic particles and their interactions with ecological factors, other particles, and chemical contaminants, 2) integrates current data and is flexible enough to incorporate new information as it becomes available, 3) considers the uncertainty inherent in our current level of understanding of nanoplastics, and 4) complies with current practices and up-to-date techniques in the field of ecological risk assessment. The Bayesian network relative risk model (BN-RRM) meets these requirements and has been a successful framework for past risk assessments involving complex contaminants at a regional scale (Landis, 2021). The BN-RRM breaks a study site down into risk regions and then uses conditional probability tables in Bayesian networks to outline causal relationships in a system and calculate relative risk (Landis, 2021). Kaikkonen et al. (2021) discusses the capability of Bayesian networks to capture the causal relationship between environmental variables using conditional probability. They are also uniquely good at integrating different types of data and capturing uncertainty in complex systems (Kaikkonen et al., 2021). The BN-RRM can incorporate new information as it becomes available and can be used in adaptive management strategies (Landis et al., 2017). This method will be useful for a nanoplastic risk assessment where our understanding is rapidly evolving, and uncertainty is high.

**FIGURE 4:**
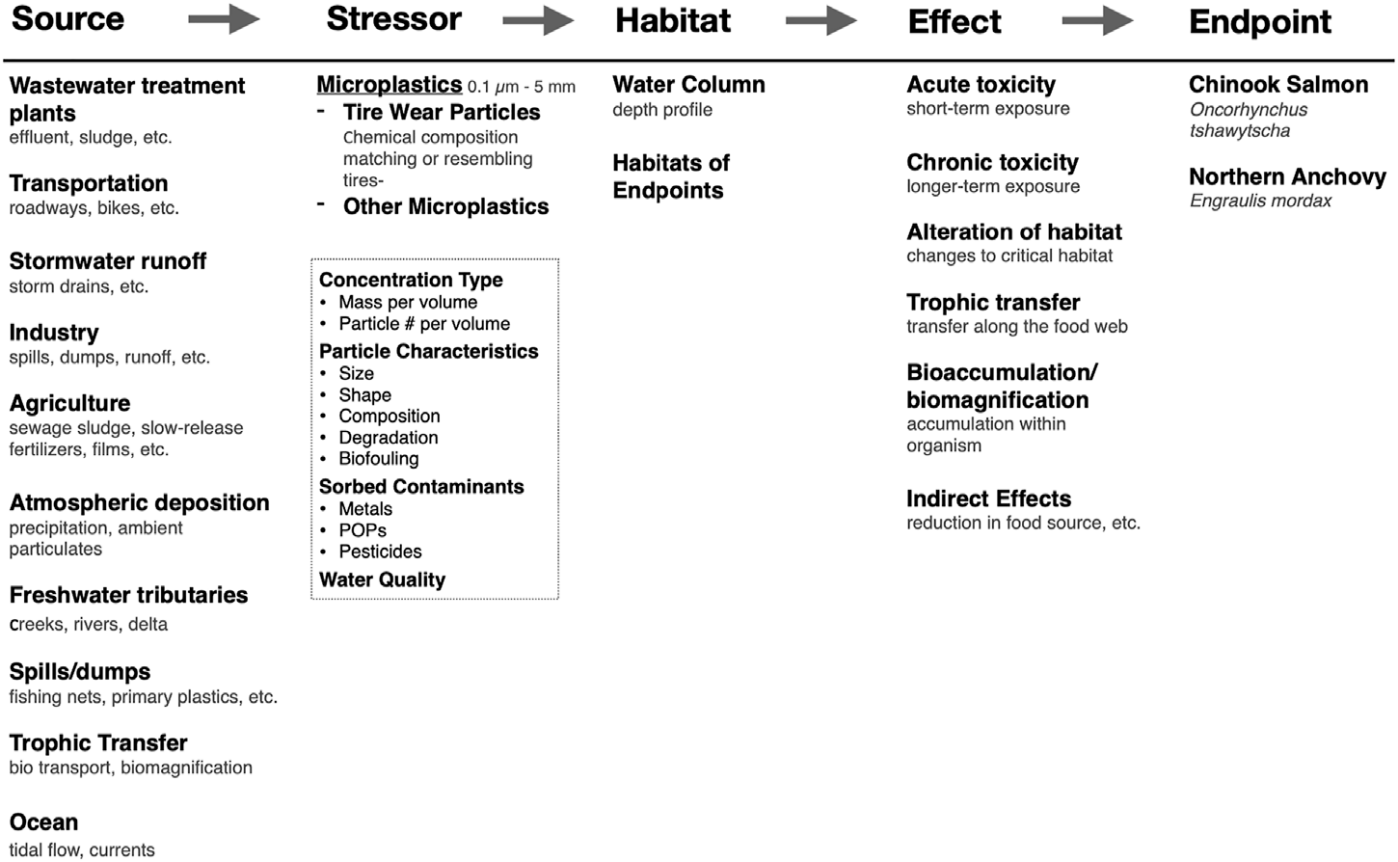
A conceptual model for an ecological risk assessment framework for microplastics and nanoplastics for the San Francisco Bay estuary. The cause-effect framework is at the top and fits the source-stressor-habitat/location-effect-endpoint/impact structure.

The critical step in the BN-RRM is the construction of a conceptual model that outlines causal relationships between the sources, stressors, habitat of chosen endpoints, toxicity, and impacts of nanoplastics. Conceptual models used for risk assessment should be specific to a region because environmental factors, management regimes, and societally valuable endpoints can vary significantly between locations. The conceptual model for the San Francisco Bay estuary is illustrated in Figure 4. This is a regional model but can be adapted to many sites and spatial scales (Landis, 2021). Sources of micro- and nanoplastics are likely similar in other urbanized sites but may vary in the magnitude of contribution. Stressors have been broken down into microplastics, nanoplastics, and tire wear particles because of differences in sources, transport mechanisms, and toxicity. In addition, particle characteristics, sorbed contaminants, and water quality parameters are included because of their potential to act as confounding stressors to the primary stressors or endpoints. Effects may be maintained between sites, but the magnitude and measured impacts may vary based on chosen endpoints, management goals, and the presence of other site-specific stressors.

### 5.3 Existing plastic risk assessments

No risk assessments have been published for nanoplastics; however, several recent publications have purported to estimate risk due to microplastics. These studies are based on models that do not meet current state of the art for ecological risk assessments that is characterized by the extensive use of probability distributions, a careful and detailed description of uncertainty, and the use of sensitivity analysis to describe key variables. Everaert et al. (2018, 2020) used a non-probabilistic approach to model concentrations of microplastics in the environment to estimate the present and future global impact given various plastic waste production scenarios (Everaert et al., 2018; Everaert et al., 2020). Adam et al. (2021) and Tamis et al. (2021) used measured rather than modeled concentration of microplastics but otherwise the methods are similar to Everaert et al. (2018, 2020). These studies concluded a low impact from microplastics however, they used a quotient method to estimate risk relying on species sensitivity distributions and predicted no-effect concentrations. Quotients are not necessarily probabilistic, the use of a single number (a quotient) underrepresents variability and may not adequately describe an exposure-response relationships (Dale et al., 2008). Coffin et al. (2022) used rescaled microplastic monitoring data from surface water samples in the San Francisco Bay and then compared these data to the management thresholds developed by Mehinto et al. (2022). Mehinto et al. (2022) proposed a microplastic monitoring framework that included four management threshold calculated using species sensitivity distributions. Coffin et al. (2022) concluded that current microplastic concentrations in some parts of the San Francisco Bay exceed risk thresholds. Unlike other studies mentioned here, they included probability distributions, a sensitivity analysis, and an uncertainty analysis but they still relied on sensitivity distributions to determine risk thresholds. Species sensitivity distributions have a number of weaknesses. They assume that the hazardous concentration for five percent of the species (HC5) is an adequate endpoint. Although widely used to approximate impact, the HC5 lacks specificity. Species sensitivity distributions also are impacted by sample number and model choice and may be limited in their ecological relevance (Fox et al., 2021).

A number of these studies also focus on global or continental-scale risk does not allow for consideration of microplastic concentrations gradients, that may exhibit patchy occurrences in the field. The patchiness of the distribution of the particles leads to an underestimate of the uncertainty in the exposure estimation. Xu et al. (2018) also attempted a risk assessment for microplastics. The study is geographically constrained, which allowed them to conduct a finer grain assessment however, they never actually calculated risk. Instead, they performed a hazard assessment to compare the relative concentrations of microplastics in the Changjiang Estuary to that of the adjacent sea. A difference in exposure does not necessarily mean an increase in risk depending on the exposure to the key endpoints specific to that habitat. A hazard assessment is a useful initial step, but it does not meet current risk assessment practice. Although attempts have been made, an ecological risk assessment using current methods has not yet been conducted for microplastics or nanoplastics.

## 6 Conclusion on data gaps to prioritize

Though existing knowledge in the microplastic and nanomaterials fields can be used to inform nanoplastic risk assessment (Table 1), there are large data gaps on nanoplastic sources, transport, and toxicological impacts that drive uncertainty to levels that make it difficult to perform a robust ecological risk assessment. While the BN-RRM can perform a robust risk assessment where data are limited, collection of data for plastic debris, specifically accounting for the nanoplastic contributions, should continue to be prioritized (Granek et al., 2020). A conceptual framework and preliminary risk assessments can help to identify these gaps helping to highlight future research needs. The data gaps specific to the needs of risk assessment are discussed in greater detail below.

Toxicity studies for nanoplastics do not provide exposure-response information suitable for modeling risk. The typical design of the current generation of toxicity tests has been screening level designs using concentrations a factor of ten apart and the use of hypothesis testing to produce single values such as no/lowest observed effect concentrations (NOEC/LOEC). This type of study design does not map the exposure-response curve, the exposure at which toxic effects are first seen, the concentration that exhibits the maximum response, and the changing slopes of the curve. In contrast, experimental designs that map the exposure-response curve provide information on the shape of the relationship, generate a mathematical description, and generate both confidence and predictive intervals to illustrate uncertainty. In addition, exposure-response curves show variability, patterns in the data, generate confidence and predictive intervals, and place a priority on the testing of more exposures to better map the relationship rather that prioritizing a null hypothesis statistical test result. The advantages described make curve-fitting the method that should be used in risk assessments (Landis and Chapman, 2011; Fox and Landis, 2016).

Quantification of exposure to microplastics and nanoplastics to a broad range of endpoints is lacking. Exposure is quantified as the probability of an endpoint being exposed to a stressor. For ecological risk assessment, it is critical to know or predict exposure concentrations so that exposure-response relationships can be used to determine toxic effects. Derivation of probability distributions for exposure requires knowledge about how nanoplastics move in the environment, where they concentrate, the rate of uptake, and physiological transport. Waldschläger and Schü;ttrumpf (2019) address part of this question by testing microplastics of various compositions, sizes, and shapes and determined settling and rising velocities for each plastic type. This information was then used in conjunction with a site-specific hydrodynamic model to develop a microplastic transport for the San Francisco Bay (Sutton et al., 2019). While this model has limitations, it is the best example of an exposure assessment. Transport mechanisms for nanoplastics likely differ in many ways from microplastics, so any transport model should be specific to nanoplastics.

A risk assessment for microplastics and nanoplastics has not been conducted but is a central priority. The assessment it will aid in determining critical data gaps to aid in making key management decisions. Any ecological risk assessment conducted for nanoplastics (1) should be site-specific, (2) consider the complex interactions that nanoplastics have with other nanoplastics, other stressors (pesticides, PCBs, metals), environmental factors (temperature, dissolved oxygen) and organisms that are exposed to them. To be a risk assessment the process be probabilistic, quantitative and descriptive of uncertainty.
