# Supplementary material for: Critical gaps in nanoplastics research and their connection to risk assessment
Source: Front Toxicol. 2023 Apr 24;5:1154538. doi: 10.3389/ftox.2023.1154538 (PMC10164945; doi:10.3389/ftox.2023.1154538)
Supplement: Supplementary file 1 [file DataSheet1.docx]

Crucial Gaps in Nanoplastic Research and their Connection to Risk Assessment

Brittany Cunningham^1^, Emma Sharpe^2^ Susanne Brander^1,3^, Wayne Landis^2^ and Stacey Harper^1,4,5*^

^1^Department of Environmental and Molecular Toxicology, Oregon State University

^2^Institute of Environmental Toxicology and Chemistry, Western Washington University

^3^Department of Fisheries and Wildlife, Oregon State University

^4^School of Chemical, Biological and Environmental Engineering, Oregon State University ^5^Oregon Nanoscience and Microtechnologies Institute;

*[Stacey.Harper@oregonstate.edu](mailto:Stacey.Harper@oregonstate.edu)

**Supplementary Material**

*Literature Search Methods:*

A literature review was conducted (October 8, 2021) using three of the most common scientific publication databases: Google Scholar, Scopus, and Web of Science. Identical search terms were used for each database to obtain an approximation of the number of total publications on macroplastic, microplastic, and nanoplastic environmental pollution. Each database was searched for publications in three size categories using the main keywords, “plastic”, “microplastic”, and “nanoplastic”. Searches were conducted for one of the main search terms plus one or more of the following additional keywords: “pollution”, “environment”, “ocean”, “marine”, “soil”, “toxic”, “toxicity”, “debris”, and “freshwater”. When the main keyword “plastic” was used (to quantify macroplastic publications), publications including the keywords “microplastic” and “nanoplastic” were excluded. The values obtained from the database searches were assessed as totals of publications in each size range, percentages of publications in each size range, and yearly publications in each size range. The goal of this review was to assess the distribution of current plastic pollution research based on particle size, and to demonstrate the relative scarcity of publications in the micro- and nano- plastic size ranges.


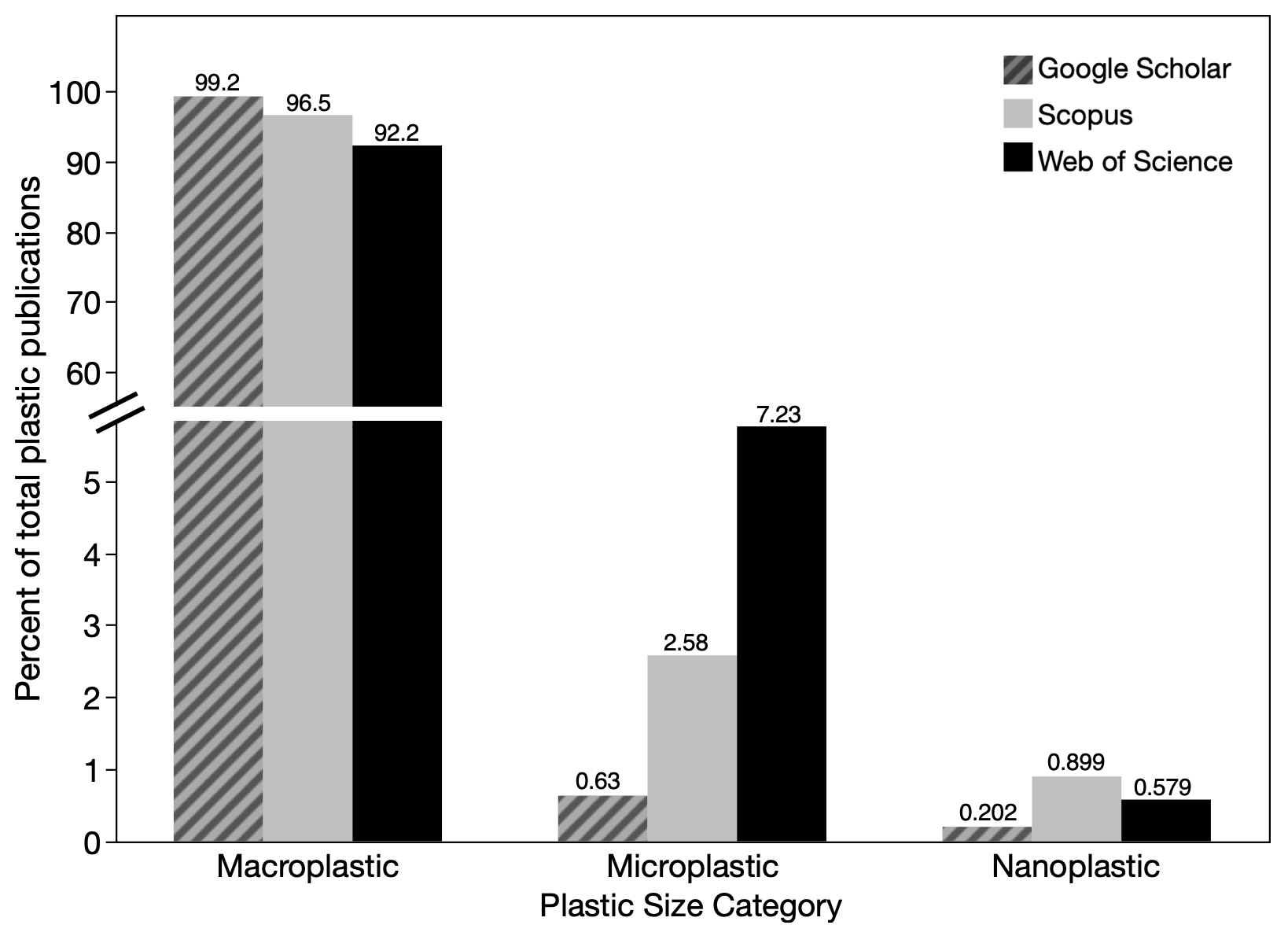


**Figure 1S**. Percentages of papers published on different size categories of plastic pollutants, based on searches of three databases (Oct. 8, 2021).


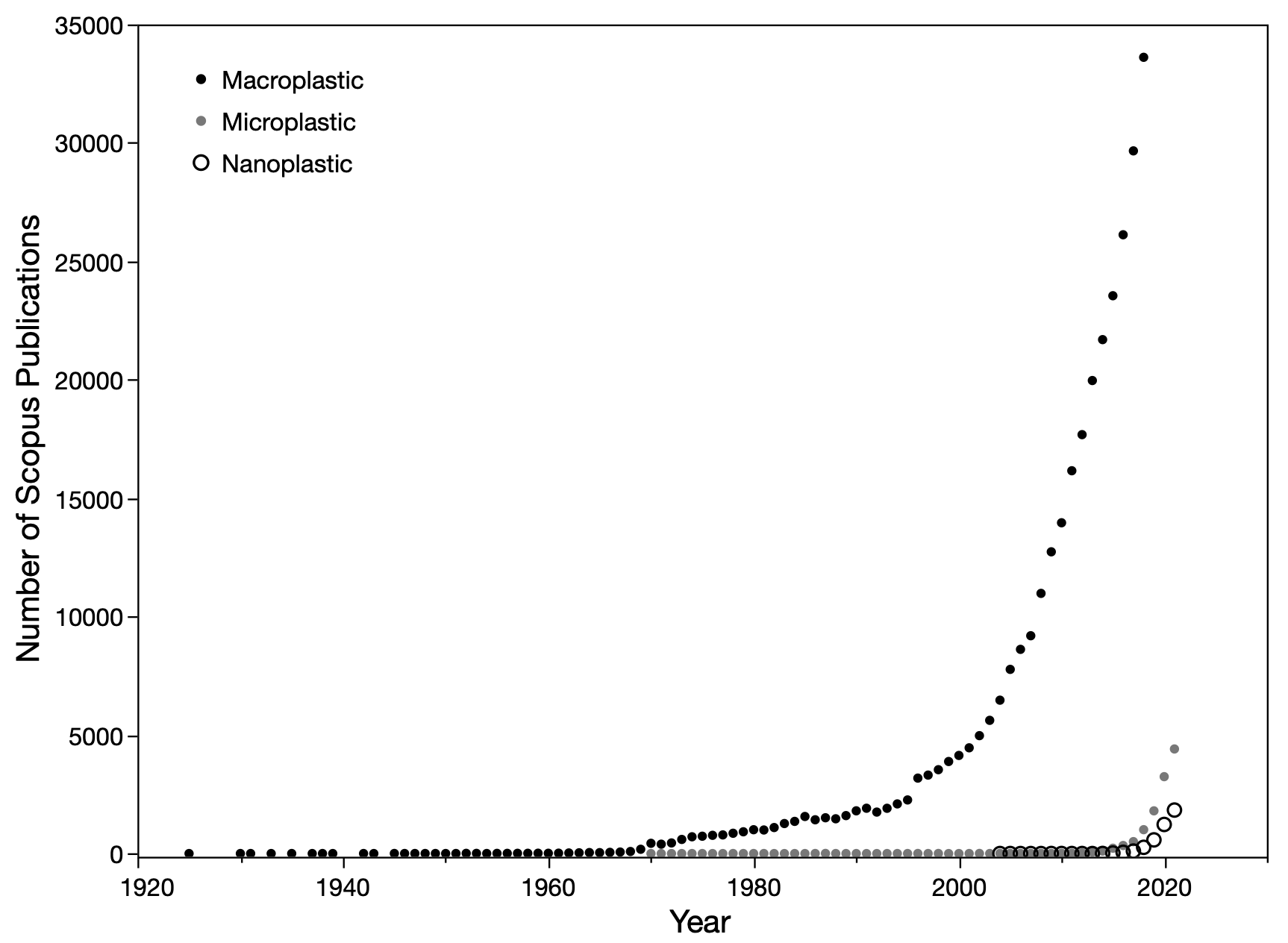


**Figure 2S**. Numbers of papers published each year on different size categories of plastic pollutants, based on a Scopus search (Oct. 8, 2021).
